# Association between Immune Checkpoint Inhibitors and Atherosclerotic Cardiovascular Disease Risk: Another Brick in the Wall

**DOI:** 10.3390/ijms25052502

**Published:** 2024-02-21

**Authors:** Linda Piras, Michela Zuccanti, Paola Russo, Francesca Riccio, Antonio Agresti, Camilla Lustri, Domenico Dardani, Armando Ferrera, Vincenzo Fiorentini, Giuliano Tocci, Giacomo Tini Melato, Massimo Volpe, Emanuele Barbato, Allegra Battistoni

**Affiliations:** 1 Department of Clinical and Molecular Medicine, Sapienza University of Rome, 00189 Rome, Italy; linda.piras@uniroma1.it (L.P.); michela.zuccanti@gmail.com (M.Z.); paolarusso9.pr@gmail.com (P.R.); francescariccio.md@gmail.com (F.R.); antonio0295@gmail.com (A.A.); cami.lustri@gmail.com (C.L.); domenicodardani1@gmail.com (D.D.); armando.ferrera@uniroma1.it (A.F.); fiorentinivincenzo94@gmail.com (V.F.); giuliano.tocci@uniroma1.it (G.T.); giacomo.tini@uniroma1.it (G.T.M.); massimo.volpe@uniroma1.it (M.V.); emanuele.barbato@uniroma1.it (E.B.); 2 IRCCS San Raffaele, 00166 Rome, Italy

**Keywords:** immune checkpoint inhibitors, cardiac event, cardiotoxicity, atherosclerosis, atherosclerotic cardiovascular events, inflammation, immunotherapy, cancer

## Abstract

In recent years, immune checkpoint inhibitors have significantly changed the field of oncology, emerging as first-line treatment, either alone or in combination with other regimens, for numerous malignancies, improving overall survival and progression-free survival in these patients. However, immune checkpoint inhibitors might also cause severe or fatal immune-related adverse events, including adverse cardiovascular events. Initially, myocarditis was recognized as the main immune checkpoint inhibitor-related cardiac event, but our knowledge of other potential immune-related cardiovascular adverse events continues to broaden. Recently, preclinical and clinical data seem to support an association between immune checkpoint inhibitors and accelerated atherosclerosis as well as atherosclerotic cardiovascular events such as cardiac ischemic disease, stroke, and peripheral artery disease. In this review, by offering a comprehensive overview of the pivotal role of inflammation in atherosclerosis, we focus on the potential molecular pathways underlying the effects of immune checkpoint inhibitors on cardiovascular diseases. Moreover, we provide an overview of therapeutic strategies for cancer patients undergoing immunotherapy to prevent the development of cardiovascular diseases.

## 1. Introduction

Cancer immunotherapy has recently brought significant changes in the field of clinical oncology, emerging as the first-line treatment for numerous malignancies such as advanced melanoma, non-small-cell lung cancer (NSCLC), small-cell lung cancer (SCLC), renal cell carcinoma and Hodgkin lymphoma (LH) which has proven to be game-changing for most of them [1,2]. Immune checkpoints, as the key regulatory molecules of immune activation, are critical to the function of the immune system. Indeed, stimulatory checkpoint pathways promote immune responses, whereas inhibitory checkpoint pathways prevent their initiation. Therefore, the rationale behind the development of immune checkpoint inhibitors (ICIs) as anticancer therapy is to trigger the activation of immune checkpoint pathways, such as programmed cell death protein 1 (PD-1) and its ligand (PD-L1) and cytotoxic T-lymphocyte-associated protein 4 (CTLA-4), allowing the host’s immune system to mount an effective T-cell-mediated immune response against tumor cells [3]. Over the last decade, ICIs have proven to be an effective therapy for the treatment of different advanced cancers. Unfortunately, due to the role of the immune checkpoint in regulating immunological tolerance and self-reactivity, it is not surprising that the use of ICIs is associated with T-cell hyperactivity towards healthy tissues, possibly leading to immune-related adverse events (irAEs). IrAEs are common in clinical practice, affecting approximately 70% of patients on ICIs, but in most cases, they are mild and easily manageable. However, severe adverse events can also occur, leading to the discontinuation of therapy and possibly affecting the overall survival (OS) of patients. Among IraEs, cardiovascular (CV) side effects can be alarming [4]. The incidence of cardiac events in patients treated with ICIs has long been underestimated due to a variety of presentations, but recently, studies have shown an incidence of major adverse cardiac events (MACEs) up to 10.3% [5,6]. Available evidence has mostly focused on the incidence of myocarditis, which is the most frequently reported adverse event, leading to the development of diagnostic and therapeutic management algorithms for this condition [5,7,8]. However, beyond myocarditis, additional ICI-related cardiotoxicities have been reported as follows: cardiomyopathy, conduction defects (heart block), atrial and ventricular arrhythmias, and pericarditis/pericardial effusion [9,10]. Recently, the association between ICI therapy and accelerated atherosclerosis, as well as the occurrence of atherosclerotic events, has been hypothesized. In light of the dramatic implementation of ICIs in clinical practice, encompassing their application in both adjuvant and neoadjuvant settings, it is crucial to gain a comprehensive understanding of how their interaction with the immune system might favor the development of atherosclerosis and MACE. This would set the basis for more precise prevention strategies and personalized treatments for patients on ICI therapy to avoid the undesirable discontinuation of effective anticancer therapies.

## 2. Methodology

This narrative review was conducted using the PubMed database: a widely recognized and comprehensive repository of biomedical literature. The search strategy involved the use of relevant medical subject headings (MeSHs), terms, and keywords to ensure a comprehensive and focused exploration of the available literature.

The primary search terms included combinations of “Immune-Checkpoint Inhibitors”, “atherosclerosis”, “cardiovascular risk”, and related synonyms. Boolean operators (AND, OR) were employed to refine the search queries and improve the relevance of retrieved articles. This search was limited to articles published in English, and no restrictions were imposed on publication dates to capture a broad spectrum of research findings. The synthesized data were qualitatively analyzed to identify common themes, patterns, and trends across the studies. The analysis also considered the potential impact of study design, sample size, and methodological differences on the reported associations.

## 3. Role of Inflammation in Atherosclerosis

Atherosclerosis is now recognized as a chronic inflammatory disease. Recent randomized controlled trials, including CANTOS (Canakinumab Anti-inflammatory Thrombosis Outcome Study) [11], COLCOT (Colchicine Cardiovascular Outcomes Trial) [12], and LoDoCO (Low-dose Colchicine) [13] have shown that the targeted application of anti-inflammatory therapies leads to a significant reduction in both atherosclerosis burden and associated adverse events. Indeed, the pathophysiology of atherosclerosis begins with the expression of cellular adhesion molecules by damaged endothelial cells, which attract monocytes to enter the sub-endothelium [3,14,15]. Once there, monocytes transform into macrophages that are polarized into different subtypes depending on their local microenvironment. Exposure to free fatty acids, oxidized lipids, and factors such as interferon-gamma (IFN-γ) promotes the pro-inflammatory phenotype. Conversely, factors like interleukin 4 (IL-4), IL-13, and IL-10 promote the anti-inflammatory phenotype. Pro-inflammatory macrophages are predominant in early atherosclerotic plaques and are responsible for intracellular lipid accumulation, foam cell formation, and the secretion of pro-inflammatory cytokines such as IL-1β and IL-6. In contrast, anti-inflammatory macrophages promote collagen formation and effective lipid clearance and are associated with atherosclerosis regression [3,16,17,18]. The hallmark of chronic inflammation in atherosclerotic plaque is the formation of a necrotic core, rich in lipids and cellular debris, resulting from the apoptosis and necrosis of foam and vascular smooth muscle cells (VSMCs). T-cell activation is also involved [3,14,15]. Indeed, during early atherosclerosis, antigen-presenting cells (APCs) present atheroma-related antigens to naïve T-cells in lymphoid tissues, leading to T-cell migration towards the plaque [3]. The T helper 1 cell (Th1) is the predominant CD4+ T-cell present in plaque, and they are strictly associated with atherogenesis due to their production of inflammatory cytokines, including IFN-γ and tumor necrosis factor-alpha (TNF-α). The production of IFN-γ enhances the recruitment of macrophages and T-cells and promotes macrophage polarization, cytokine secretion, and foam cell formation [3,18]. IFN-γ is also thought to inhibit the proliferation of VSMCs, thereby contributing to decreased plaque stability. The atherogenic role of IFN-γ is clearly demonstrated. In an experimental study, mice with dysfunctional IFN-γ receptors developed smaller and more phenotypically stable atheroma compared to the controls [19]. TNF-α promotes atherosclerosis through leukocyte recruitment, inflammatory cytokine production, and the promotion of endothelial cell damage and oxidative stress. TNF-α deficient mice have been shown to exhibit smaller plaque lesions, whereas the presence of TNF-α is associated with increased lesion necrosis and more advanced plaque progression in mice [20]. The inhibition of Th1 differentiation in mice is also atheroprotective and reduces the amount of IFN-γ detected in plaques [18,21,22,23,24,25].

The atheroprotective role of T regulatory cells (Treg) has been largely investigated. These effects are primarily mediated through the secretion of Transforming Growth Factor-B (TGF-β) and IL-10. TGF-β inhibits the recruitment and activation of T-cells and macrophages and increases plaque stability by promoting VSMC proliferation [26]. Mice with defective TGF-β receptors have larger atherosclerotic lesions with increased T-cell and macrophage composition, increased IFN-γ expression, and a more vulnerable plaque phenotype [23]. IL-10 reduces Th1 differentiation and prevents the recruitment and cytokine secretion of T-cells and macrophages. An experimental study with IL-10-deficient mice demonstrated increased susceptibility to atherosclerosis and higher T-cell infiltration and IFN-γ expression compared to the controls [25]. In addition, the Treg secretion of IL-10 promotes the transformation of macrophages from the pro-inflammatory to the anti-inflammatory phenotype, potentiating its atheroprotective effect [24]. Ait-Oufella et al. demonstrated that deficiency in Treg was associated with increased plaque size and a more advanced plaque phenotype in mice and that the subsequent co-transference of Treg reduced inflammatory cell infiltration and plaque size [27]. In addition, the number of Treg cells was inversely correlated with plaque vulnerability in human carotid arteries [28].

The roles of the remaining T-cell subtypes in atherosclerosis are less well-defined. Th2 cells, by decreasing the production of IFN-γ, are thought to have an atheroprotective role. This has been further supported by the fact that the deficiency in IL-5, one of the primary Th2-secreted cytokines, has been shown to accelerate atherosclerosis development in mice [29,30]. On the other hand, the deletion of another Th2 cytokine, IL-4, has been shown to decrease plaque lesion size in mice [31]. Thus, the role of Th2 cells in atherogenesis remains debated.

**Figure 1:**
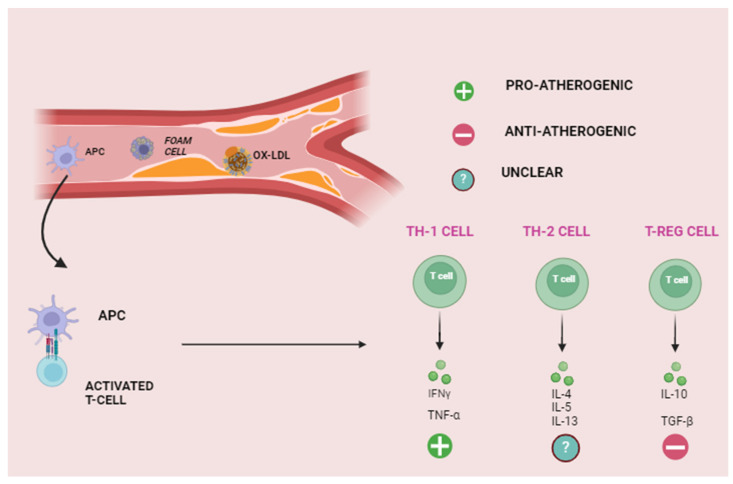
Role of inflammation in atherosclerosis. APC, antigen-presenting cell; OX-LDL, oxidized low-density lipoprotein; TH, T-helper lymphocite; T-Reg; and T-regulatory lymphocyte.

Th17 cells have received considerable attention for their role in atherogenesis in recent years. A study analyzing human coronary atherosclerotic plaque demonstrated that IL-17, the principal cytokine released by Th17, plays synergistically with IFN-γ to promote inflammation by increasing the secretion of IL-6 [32]. However, the cytokine secretion of Th17 cells relies on an environmental context, making the role of Th17 cells in atherosclerosis difficult to define. Several studies have indicated an atherogenic role in IL-17 [33], while others suggest an atheroprotective role by promoting plaque stability via type I collagen production [34,35]. Despite these inconclusive findings, the ratio between Th17 and Treg cells has been implicated in atherosclerosis progression, with increased Th17 and decreased Treg levels observed in patients with coronary atherosclerosis. Recent data suggest that the inhibition of CD69, a key molecule regulating T-cell differentiation, leads to elevated Th17/Treg ratios and the exacerbation of atherosclerosis in mice [35]. Therefore, the delicate balance between T-cell subsets might also have crucial implications on autoimmune conditions and off-target adverse events related to ICI therapy (Figure 1).

## 4. Immune Checkpoints and Pathophysiology of ICI-Related CVD

Immune checkpoint receptors such as PD-1, CTLA-4, and lymphocyte-activation gene 3 (LAG-3) are expressed on the surface of T-cells preventing T-cell activation [36]. Indeed, T-cell activation occurs due to the binding of the T-cell receptor (TCR) to major histocompatibility complex I or II (MCH-I/II) and additional co-stimulation through CD28-CD80/CD86 binding, which results in the recruitment of multiple molecules, including phosphoinositide 3-kinase (PI3K), to the intracellular part of CD28 [37]. PI3K recruitment activates the PI3K/protein kinase B (Akt) pathway, which promotes proliferation, differentiation, and anti-apoptotic signaling in T-cells [38]. The activation of T-cells results in the differentiation of CD8 cells into cytotoxic T-cells and CD4 cells into stimulatory Th cells or inhibitory Treg cells, depending on the cytokines in the environment [39]. On the other hand, inhibitory immune checkpoint receptors can prevent the over-activation of the immune system and promote self-tolerance. PD-1 is expressed on the surface of T-cells and interacts with two ligands, PD-L1 and PD-L2 [40]. PD-L2 is mainly expressed in macrophages and dendritic cells, whereas PD-L1 is present in hematopoietic cells and tissue cells in various organs [3]. The binding of PD-1 to either of its ligands leads to down-regulated T-cell activity through downstream SHP-2 signaling and the subsequent dephosphorylation and inhibition of the downstream PI3K-Akt pathway, resulting in decreased inflammatory cytokine production, cell survival signals, and proliferation [41,42]. It has been suggested that the PD-1 suppression of T-cells normally takes place at later stages of an immune response in peripheral tissues [43].

CTLA-4 is located intracellularly and translocated to the surface upon T-cell activation [41]. It binds to CD80 and CD86 and suppresses T-cell activation through PI3K downstream signaling inhibition, similar to PD-1 inhibition [41,44]. Additionally, CTLA-4 can interact with protein phosphatase 2A (PP2A), which dephosphorylates AKT, quenching the pathway further [45]. This reduces cytokine production in CD8 T-cells and promotes the differentiation of CD4 T-cells towards Treg cells. In contrast to PD-1, CTLA-4 suppresses T-cell activation at an earlier stage of the immune response [43].

LAG-3 is expressed on the surface of activated T-cells and constitutively on Treg cells. It is involved in suppressing T-cell expansion, increasing cell death and Treg function. The receptor is homologous to CD4 and can bind MHC-II with higher affinity [46]. Besides MHC-II, additional ligands for LAG-3 include liver sinusoidal endothelial cell lectin (LSECtin), Galectin-3 (Gal-3), and fibrinogen-like protein 1 (FGL1) [47]. LAG-3 intracellular signaling mechanisms remain largely unknown, but an association with the inhibition of the TCR/CD3 activating pathway has been suggested, resulting in reduced T-cell expansion and the cytotoxic activity of CD8 cells [48].

The inhibition of immune checkpoints exploited in oncological therapy can lead to the development of a broad spectrum of CV irAEs, such as myocarditis, conduction blocks, ventricular arrhythmias, pericarditis, and ventricular dysfunction [4]. The underlying pathophysiological mechanisms still remain unclear [49]. Cases of severe myocarditis due to lymphocytic infiltration in mice deficient in CTLA-4 and PD-1 have been shown in preclinical studies. Moreover, the disruption of the PD-1 gene may lead to the sudden death of mice due to the development of severe heart failure secondary to dilated cardiomyopathy [49]. Other studies have also shown that immune checkpoint dysregulation may lead to medium and large vessel vasculitis. A defective upregulation of PD-L1 in the dendritic cells of the vessel wall leads to the unopposed activation of PD-1 positive T-cells and the production of inflammatory cytokines, thereby contributing to the development of giant cell arteritis [50]. However, data in this regard are still lacking. While the molecular mechanisms underlying myocarditis, cardiomyopathy with ventricular dysfunction, and vasculitis appear to involve the abnormal lymphocytic infiltration of the myocardium and vascular endothelium with subsequent inflammatory response, the mechanisms underlying arrhythmias, pericarditis, and conduction blocks are less understood [49].

Recently, emerging preclinical and clinical evidence (Table 1) has shown an association between ICIs with the progression of atherosclerosis and the development of MACE [3]. Indeed, immune checkpoint proteins are also important negative regulators of atherosclerosis [51]. PD-1 and PD-L1 play a role in suppressing T-cell-driven inflammation within plaques, and lower levels of the PD-1/PD-L1 pathway have been associated with an increased coronary atherosclerotic plaque burden in preclinical models. Studies on PD-L1-deficient mice showed elevated atherosclerotic burden and increased numbers of CD4+ and CD8+ T-cells in the lesions. These mice also exhibited larger lesions with abundant CD8+ T-cells and macrophages. Experimental studies have shown that PD-L1^−/−^Ldlr^−/−^ cells were more susceptible to APC-induced proliferation and expressed higher levels of proatherosclerotic cytokines such as IFN-γ and TNF- α [52,53]. In hyperlipidemic mice, both PD-L1/PD-L2 deficiency and PD-1 receptor inhibition are associated with increased atherosclerotic lesion size, increased plaque T-cell activation, and enhanced TNF-α secretion [52,53]. In contrast to CTLA-4 inhibition, PD-1 inhibition also exhibited an increased macrophage content and enhanced cytotoxicity of CD8+ T-cells in plaques [53]. Furthermore, human studies demonstrated reduced PD-1 and PD-L1 expression on T-cells and myeloid dendritic cells in patients with coronary artery disease (CAD) [54]. These findings highlight the critical role of the PD-1/PD-L1 pathway in down-regulating proatherogenic T-cell responses and limiting atherosclerosis by controlling APC dependent T-cell activation.

In addition, a recent study conducted by Michel et al. [55] has provided new valuable insights into the PD-1/PD-L1 dyad. Specifically, the authors demonstrated a high expression of PD-L1 in the myocardium, predominantly expressed by vascular endothelial cells. This evidence suggests that ICI-mediated atherosclerosis is not solely a consequence of drug-induced systemic inflammation but might involve the direct infiltration of lymphocytes into the myocardium facilitated by vascular endothelial cells. In this study, the authors found an augmented density of lymphocytes in the myocardium of mice treated with ICIs [55]. Interestingly, to assess whether the observed infiltration of lymphocytes was associated with changes in LV function, a functional assessment was conducted. The authors found that mice treated with ICIs exhibited a reduction in left ventricular ejection fraction (LVEF) both at rest and after ionotropic stress [55]. These findings suggest that the cardiotoxicity induced by ICI therapy extends beyond well-known side effects such as myocarditis. It can be inferred that myocardial inflammation might manifest in various forms and degrees. Notably, acute manifest cardiotoxicity may be observed only in a subset of patients, while subtler cardiotoxic effects exert significant detrimental effects over extended time periods [55]. Moreover, recent evidence derived from pre-clinical models has demonstrated that ICIs, particularly anti-PD1 agents, also have an impact on ischemia–reperfusion injury [56]. The authors showed that PD-1 depletion in mice led to the establishment of intracardiac baseline inflammation and increased endothelial injury in vivo. Moreover, immunofluorescence imaging showed decreased PD-L1 expression in the infarct zone, highlighting the involvement of PD-L1 in myocardial response to injury. The decreased expression of PD-L1 in the infarcted area seems to support an inflammatory reaction with subsequent remodeling and tissue healing. In addition, the authors found that the pharmacological depletion of PD-1 prior to reperfused acute myocardial infarction did not alter the area of infarction but led to increased numbers of CD8+ T-cells in treated mice. It can be hypothesized that PD-1/PD-L1 signaling plays a significant role during myocardial injury, so patients affected by myocardial infarction and receiving ICIs might be at risk for adverse outcomes [57].

The over-expression of CTLA-4 in T-cells, either through transgenic models or systemic treatment with abatacept, a CTLA-4–immunoglobulin (Fc region of IgG1) fusion protein, causes a reduction in atherosclerotic lesion formation. Conversely, inhibiting CTLA-4 using antibodies or the dual inhibition of CTLA-4 and PD-1 accelerates atherosclerosis with increased T-cell-driven endothelial inflammation and plaque area. Therefore, the CTLA-4 pathway appears to be crucial in modulating atherosclerosis [58,59]. Ewing et al. showed that the use of abatacept prevented CD4+ cell activation and reduced atherosclerosis development in murine femoral arteries by 78%, whereas the administration of CTLA-4 blocking antibodies increased atherosclerotic lesion sizes [60]. Similarly, mice with a decreased membrane expression of CTLA-4 had a greater quantity of atherosclerotic plaque, whereas pretreatment with abatacept seemed to stabilize plaques [61]. Moreover, Poels et al. evaluated the effect of antibody-mediated CTLA-4 inhibition on atherogenesis. The authors demonstrated a two-fold increase in atherosclerotic lesion size in mice treated with CTLA-inhibiting antibodies. This was primarily mediated by the activation of T-cells. CTLA-4 inhibition was also associated with plaque progression to more unstable phenotypes, with decreased collagen content and larger necrotic core areas [59].

A substudy of the Multiethnic Study of Atherosclerosis (MESA) study showed that subjects with CAD exhibited higher levels of LAG-3. Furthermore, this study established LAG-3 as a significant predictor of the risk of developing CAD. However, additional studies are needed to investigate whether increased LAG-3 expression might play a contributory or compensatory role in atherosclerosis [62,63].

**Figure 2:**
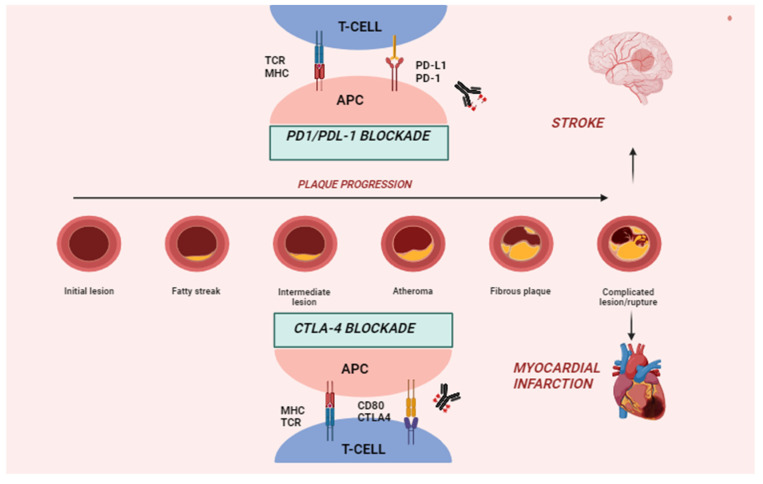
Role of ICIs in plaque progression and atherosclerotic cardiovascular events. APC, antigen-presenting cell; MHC, major histocompatibility complex.

In summary, experimental models of atherosclerosis have provided insights into the role of the PD-1/PD-L1 dyad and CTLA-4 in suppressing T-cell-driven inflammation, thereby limiting plaque development, progression, and instability. Consequently, the inhibition of these pathways due to immunotherapies for cancer treatment may inadvertently activate T-cells within atherosclerotic plaque and potentially accelerate atherosclerosis in these patients (Figure 2).

Novel classes of immune therapies also include the macrophage-mediated immune checkpoint CD47. CD47 is an immunoglobulin-like molecule that binds to signal regulatory protein alpha (SIRPα) and impairs phagocytosis. In tissues with high rates of apoptosis and cell turnover, such as within tumors and atherosclerotic necrotic cores, the effective clearance of apoptotic cellular debris helps prevent further inflammatory response [64]. This process, known as “efferocytosis”, refers to the programmed cell removal by which macrophages detect cell surface markers that can start phagocytosis, collectively termed “eat me” signals [65]. By contrast, cells may express markers that impair phagocytosis, known as “do not eat me” signals, such as CD47. Kojima et al. [66] demonstrated elevated CD47 expression in murine and human atherosclerotic plaques, particularly within the necrotic core. Treatment with CD47 inhibitory antibodies in atherosclerotic models significantly reduced atherosclerosis by enhancing efferocytosis, leading to the improved removal of damaged vascular smooth muscles and macrophages in vivo. Furthermore, CD47 inhibition downregulated genes associated with macrophage response to IL-1 and IFN-γ, resulting in the decreased inflammation observed with positron emission tomography/computed tomography (PET/CT) imaging within atherosclerotic plaques. Moreover, CD47 has been implicated in diminishing the macrophages’ ability to clear opsonized targets, including the opsonized clonal smooth muscle cells believed to be a major component of atherosclerotic plaque [66,67]. In recent years, CD47 inhibitor therapies have been tested in oncological clinical trials with the aim of increasing tumor cell recognition and phagocytosis by macrophages. Magrolimab, the first-in-class anti-CD47 antibody, showed promising results in Phase 1B trials of relapsed and refractory non-Hodgkin’s lymphoma [68]. It recently gained breakthrough therapy designation by the Food and Drug Administration, and ongoing trials are evaluating its efficacy in various hematologic and solid tumor malignancies. Interestingly, a small retrospective analysis of non-Hodgkin’s lymphoma participants demonstrated a reduction in FDG uptake in the carotid arteries after 9 weeks of Magrolimab treatment, suggesting that CD47 inhibition might reduce vascular inflammation [66]. Thus, CD47 inhibition may counteract oncogenesis as well as atherogenesis. Treg cell activity can influence macrophage efferocytosis. Proto et al. [69] showed that Treg cell expansion enhanced efferocytosis via the expression of IL-13. PD-1 and CTLA-4-targeted ICI therapies have been suggested to suppress Treg activity. Therefore, by decreasing efferocytosis, they can exacerbate atherosclerosis. However, extensive research is needed to confirm this link.

**Table 1:** Preclinical studies on the role of CTLA-4, PD-1, and PD-L1/2 in atherosclerosis.

Models	Atherosclerosis	Effect on Plaque
Pdl1/2^−/−^ Ldlr^−/−^ mice [52]	↑	↑ CD4, CD8, macrophages
Pd1^−/−^ Ldlr^−/−^ mice [53]	↑	↑ CD4, CD8, macrophages and apoptotic cells
Antibody-mediated PD-1 inhibition in Ldlr^−/−^ mice [53]	↑	↑ CD4, CD8
Anti-CTLA-4 antibody in ApoE3 Leiden mice [60]	↑	↑ intimal thickening and intimal leukocytes
Anti-CTLA-4 antibody in Ldlr^−/−^ mice [59]	↑	↑ advanced lesions, necrotic core and CD3 cells
Combined anti-CTLA-4 and anti-PD-1 antibodies in Ldlr^−/−^ mice [60]	↑	↑ CD3, CD8, advanced lesions, necrotic core and apoptotic macrophages

CTLA-4: Cytotoxic T-lymphocyte-associated protein 4; PD-1: programmed cell death protein 1; and PD-L1: programmed cell death protein ligand 1; PD-L2: programmed cell death protein ligand 2; Up arrows indicate an increase in atherosclerotic burden.

## 5. Cholesterol Metabolism and T-Cell Function in Cancer

In clinical practice, the association between obesity, metabolic syndrome, dyslipidemia, and higher cancer risk is well-established [3]. In particular, modifications in lipid metabolism may influence the immune system’s response to tumor activity. Cholesterol is essential to cancer progression, playing a pivotal role in the formation of cellular membranes during rapid proliferation, tumor migration, and invasion. Metabolic syndrome features, including hypertriglyceridemia, hyperglycemia, and hypercholesterolemia, contribute to a chronic inflammatory state that paradoxically hampers physiologic immune functions [3]. This stimulates the production of myeloid-derived suppressor cells (MDSCs) through a phenomenon termed “emergency myelopoiesis” [70]. MDSCs block T-cell functions via alterations in TCR receptors and through the secretion of TGF-β, IL-10, and cytokines, leading to reduced T-cell function. In a study on atherosclerotic Apoe^−/−^ mice, Ma et al. demonstrated the negative effect of hypercholesterolemia on IL-9 levels, impairing CD8+ T-cell differentiation and antitumor response [71]. Higher levels of cholesterol in the tumor microenvironment induce T-cell exhaustion and the expression of immune checkpoints such as PD-1, TIM-3, and LAG-3 on CD8+ T-cells. Given the interplay between hypercholesterolemia and T-cell function, it is consequent that cholesterol levels may modulate a patient’s responses to immunotherapy. Perrone et al. observed that, among patients receiving ICIs, baseline hypercholesterolemia correlated with OS rates [72]. Additional studies have established a more favorable prognosis in obese patients treated with ICIs [73]. Consequently, it can be postulated that cancer patients with high cholesterol levels may experience exacerbated T-cell dysfunction through these aforementioned mechanisms, with a subsequent more pronounced response to the restoration of T-cell function by ICIs. However, data are still limited to date, and whether hypercholesterolemia might enhance ICI responses or simply function as a biomarker of chronic inflammation, rendering patients more susceptible to immune restoration remains to be established [3].

## 6. ICIs Therapy and Atherosclerotic Cardiovascular Disease: Clinical Implications

Based on preclinical evidence, recent studies have investigated whether ICI therapy might be associated with atherosclerosis and atherosclerotic cardiovascular diseases (ASCVDs). Most evidence in this setting derives from single-center observational studies and meta-analyses [9,74,75,76,77,78] (Table 2).

In a retrospective analysis of clinical trials involving ICIs such as nivolumab, pembrolizumab, atezolizumab, avelumab, and durvalumab in patients affected by NSCLC, Hu et al. reported a 1% occurrence of myocardial infarction and a 2% incidence of stroke [9]. Additionally, Oren et al. conducted a single-center registry study of 3326 patients with various malignancies treated with ICIs, including atezolizumab, avelumab, ipilimumab, nivolumab, and pembrolizumab, revealing a 7% occurrence of both myocardial infarction and stroke within a 16-month period [77].

Calabretta et al. [79] used 18-FDG-positron emission tomography to quantify atherosclerotic inflammatory activity in twenty patients with melanoma undergoing ICI treatment over a 4-month period. FDG uptake significantly increased in non-calcified and mildly calcified segments, indicating increased atherosclerotic inflammatory activity in early lesions after ICI therapy. These findings suggest that ICI therapy may influence local innate immune cells and aggravate inflammation, particularly in early non-calcified and mildly calcified atherosclerotic lesions.

In the most extensive single-center study conducted to date, Drobni et al. compared 2842 patients receiving various ICIs (primarily PD-1 inhibitors) with controls matched for age, cancer type, and CV history, assessing the risk of ASCVD-related events over a 2-year follow-up. The use of ICIs was associated with a more than four-fold increase in the risk of ASCVD (HR 4.7, (95% CI 3.5–6.2, *p* < 0.001), a seven-fold higher risk of myocardial infarction (HR 7.2 [95% CI 4.5–11.5, *p* < 0.001]), a three-fold increase in the risk of coronary revascularization (HR 3.3, [95% CI 2.0–5.5], *p* < 0.001), and a four-fold increase in the risk of stroke (HR 4.6 [95% CI 2.9–7.2], *p* < 0.001) [74]. Laenens et al. also conducted a study involving more than 600 patients who received ICI treatment, with a median follow-up of 13 months. They reported a 10.3% incidence of ASCVD, which included myocardial infarction, heart failure, and cerebral ischemia. The overall mortality was 54.9%, with a CV death rate of 1.9% [6]. In a recent case–control study conducted on patients with lung cancer, Drobni et al. found that there was a higher rate of aortic atherosclerotic plaque progression in patients who received ICI therapy [80]. Consistent with the findings from Calabretta et al., [79] non-calcified plaque extremely progressed, suggesting that these patients may be at a higher risk of plaque rupture. Among these cases, those who received combined ICI therapy had higher progression rates than those who received single-agent therapy.

A recent study [81] showed that patients treated with ICIs had a two-fold higher atherothrombotic (ATE) risk compared to controls (RR, 2.01 [95% CI (1.61–2.51)]; *p* < 0.001). The subgroup analysis revealed no significant increase in ATE risk for ICI-treated patients within 1 year, whereas the ATE risk rose by 41% at 1 year (*p* = 0.010) and 97% at 4 years (*p* ≤ 0.001) in the ICI group.

Taken together, these results provide convincing data to consider ICI therapy as a modifier of atherosclerosis and CV risk [80]. However, the association between the use of ICIs and atherosclerosis has mainly been suggested by smaller observational studies. Further evidence with larger sample sizes and longer study durations is needed to confirm and better estimate the association between ICI use and atherosclerosis.

## 7. Pharmacotherapy for Cardiovascular Risk Reduction in Cancer Patients Treated with ICIs

Considering the extant evidence, it is recommended that cancer patients eligible for ICIs undergo meticulous evaluation for established CV risk factors utilizing user-friendly scoring systems such as the Systemic Coronary Risk Estimation (SCORE) [82]. The baseline evaluation and regular monitoring of body weight, blood pressure, cholesterol levels, and glycemic status are imperative for all cancer patients undergoing treatment with ICIs. It is advisable to optimize the management of CV risk factors and initiate appropriate medical therapy for primary or secondary prevention prior to, during, and following ICI therapy. Patients should be actively encouraged to quit smoking and adopt a healthy lifestyle and diet regimen, recognizing the challenges inherent in adhering to regular physical activity and strict dietary measures, particularly among cancer patients, especially those with metastatic disease [83]. Furthermore, it is essential to provide comprehensive patient education regarding the potential for CV adverse events during ICI therapy. This is paramount for improving patient compliance with CV treatment regimens and fostering awareness of CV symptoms that may frequently go unnoticed, resulting in delayed diagnoses. Encouraging patients to undergo more frequent cardiac assessments and monitoring, alongside adopting healthier lifestyles, can aid in the early detection and management of potential CV effects associated with ICI therapies.

In addition to behavioral changes, drugs with known cardio-protective properties have been tested in this setting.

**Figure 3:**
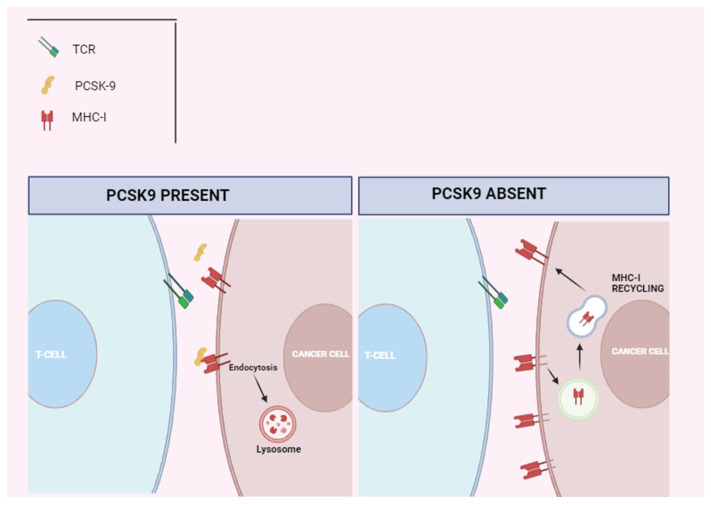
PCSK9 impedes the recycling of MHC-I to the cell surface by facilitating MHC-I degradation. MHC-I, major histocompatibility complex I; PCSK-9, proprotein convertase substilisin/kexin type; and TCR, T-cell receptor.

Statins, beyond their established cholesterol-lowering properties, have demonstrated pleiotropic effects, including the ability to stabilize plaque, reverse endothelial dysfunction, and mitigate inflammation. A retrospective analysis showed that statins can modulate ICI-induced atherosclerosis. Indeed, patients on statins had a lower rate of atherosclerotic progression compared to those not on statins. Despite this, statin therapy did not reduce the risk of adverse CV events in this study [74]. Moreover, statins are known to be associated with a significant risk of muscle damage, as evidenced by a sub-analysis of Drobni et al.’s study [80] Indeed, patients on statins showed a two-fold increased risk of developing myositis, a known adverse event also associated with ICIs. This might limit the use of statins in patients on ICIs. Consequently, the effect of alternative lipid-lowering agents, such as proprotein convertase substilisin/kexin type 9 (PCSK9) inhibitors, on atherosclerosis and atherosclerotic events has been investigated. PCSK9 inhibitors have been found to reduce LDL cholesterol levels and decrease ASCVD independently of cholesterol reduction [84,85]. A study conducted by Liu et al. in patients undergoing ICI therapy showed that the use of PCSK9 inhibitors can also enhance the effectiveness of immunotherapy by up-regulating the expression of MHC-I on tumor cells [86]. Indeed, PCSK9 prevents the recycling of MHC-I to the cell surface by facilitating MHC-I degradation (Figure 3). The inhibition of PCSK9 results in an augmented MHC-I expression on the tumor cell surface, thereby facilitating the infiltration of cytotoxic lymphocytes within the tumor microenvironment [87]. PCSK9 directly interacts with amyloid precursor-like protein 2 (APLP2), which acts as a molecular bridge that facilitates the lysosomal degradation of MHC I [88]. As a result, the use of PCSK9 inhibitors can systematically induce a peripheral immune response against tumor cells via optimizing their recognition by T lymphocytes [89]. The downregulation of MHC-I expression is a known strategy employed by tumor cells to evade immune surveillance and has been associated with resistance to immunotherapy in cancer patients. Preclinical studies in mice with melanoma also suggested that silencing the PCSK9 gene significantly enhanced the response to ICIs [90,91]. Notably, PCSK9 is not only expressed by cancer cells but is also released by them [92,93]. A study in NSCLC patients showed that low systemic levels of PCSK9 (<95 ng/mL) predict a more favorable response to the PD-1 inhibitor Nivolumab and are associated with improved OS compared to patients with higher levels (>120 ng/mL) [94]. In a recent biochemical study, aptamer PL1 and PCSK9 siRNA were found to enhance anti-PD-1/PD-L1 therapies in human colorectal cancer cells by increasing the expression of IFN-γ and Granzyme B [86]. Furthermore, the reduction in total cholesterol levels leads to the inhibition of the ACAT-1 enzyme, responsible for cholesterol esterification, further enhancing the anti-tumor immune response [95]. Therefore, combining PCSK9 inhibitors with ICIs may not only reduce the risk of ASCVD but also improve the efficacy of antitumor therapy, particularly in patients who exhibit poor response due to reduced MHC-I expression [87].

Moreover, in recent studies, it has also been demonstrated that PCSK9 plays a regulatory role in proliferation and apoptosis in human cancer cells [96]. For instance, in conditions such as neuroglioma and NSCLC, the silencing of the PCSK9 gene has been shown to activate cancer cell apoptosis through pathways involving caspase-3 and XIAP/p-Akt [97]. In the context of colon cancer, a study has revealed that PCSK9 expression is upregulated in tumor cells and correlates with their invasiveness. The downregulation of the PCSK9 gene reduces the colonic epithelial-to-mesenchymal transition, n-cadherin, and type 9 metalloproteases through the PI3K/AKT pathway. Additionally, PCSK9 is found to regulate the polarization of colonic peritumor macrophages, resulting in a pro-inflammatory and pro-metastatic phenotype [98].

Based on these findings, drugs inhibiting PCSK9 seem to potentially serve as crucial cardioprotective strategies for mitigating ASCVD in cancer patients undergoing ICI therapy. The treatment with PCSK-9 inhibitors might translate into the enhancement of immunotherapy efficacy, the inhibition of mechanisms that confer resistance to apoptosis, a reduction in the risk of destabilization of atherosclerotic plaques, and possibly a reduction in ASCVD [87].

Immunosuppressive therapy represents a conventional approach for managing cardiac irAEs. A study conducted by Drobni et al. [80] among patients undergoing ICI therapy revealed a lower annual rate of plaque progression in individuals treated with corticosteroids compared to those not receiving corticosteroids (3.5% vs. 6.9%, *p* = 0.04). However, it is important to note that immunosuppressive medications may attenuate the anticancer efficacy of ICIs [55]. Therefore, there is a pressing need for preventive strategies aimed at mitigating the development of cardiotoxicity without compromising the anticancer effectiveness of ICIs, rather than relying solely on salvage therapy for acute cardiotoxic events. Recently, Michel et al. [55] demonstrated that anti-PD-1 therapy resulted in elevated TNFα levels in the myocardium of mice treated with ICIs. Utilizing a TNFα blockade regimen preserved left ventricular (LV) function while maintaining the anticancer efficacy of ICIs. This indicates that the TNFα blockade could function as a preventive strategy to mitigate clinically evident cardiac complications while sustaining the anticancer effectiveness of ICIs. This observation is consistent with previous evidence, illustrating that the TNFα blockade ameliorates ICI-induced colitis in mice subjected to dual checkpoint inhibition. Insights into the mechanisms underlying sustained anticancer efficacy can be gleaned from early preclinical and clinical findings.

At the immune signaling level, TNFα has been observed to induce the upregulation of secondary immune checkpoints, including TIM3 and PD-L1, within cancer cells, consequently diminishing the efficacy of anti-PD1 therapy. However, the blockade of TNFα prevents these alterations, thereby augmenting the effectiveness of anti-PD-1 therapy [99]. Inhibiting TNFα reduces the anti-PD-1-induced cell death of tumor-infiltrating lymphocytes, thus amplifying the anticancer immune response. While tumor-specific mechanisms may counteract the immunosuppressive effects of TNFα inhibition during ICI therapy, a substantial systemic immunosuppressive effect persists, likely influencing cardiac immunity.

## 8. Limitations of Data So Far and Future Perspectives

Preclinical investigations have yet to fully elucidate how alterations in immune checkpoints may impact atherosclerosis [3,100]. Furthermore, murine models, commonly employed in preclinical studies involving ICIs, may not be entirely reliable. This is due to the fact that most ICIs do not cross-react with murine epitopes. Additionally, there are significant differences between the immune systems of mice and humans in terms of immune cell types, their distribution, and the expression of certain immune-related molecules, which can influence the efficacy and safety profile of ICIs [101]. Specifically, ICIs may exhibit differing epitope specificities in mice compared to humans, resulting in variations in the binding affinity and downstream signaling pathways activated by these antibodies [102]. Therefore, caution is warranted when extrapolating findings from murine studies to human clinical trials [103].

At the clinical level, the potential link between ICIs usage and atherosclerosis has been hinted at by small observational studies. Thus, additional evidence from studies with larger sample sizes and longer durations is imperative to precisely gauge the incidence of adverse CV events within this population [3].

The incorporation of both traditional and innovative imaging modalities for the early detection of atherosclerosis in patients receiving ICIs could prove valuable in identifying and quantifying the burden and advancement of atherosclerotic plaques in this high-risk cohort. Specifically, CT imaging, including the possible utilization of coronary CT angiography, shows the potential for longitudinally assessing the progression of atherosclerotic plaque in individuals undergoing ICI treatment [100].

Recently, molecular imaging studies have introduced the use of labeled antibodies as immune-PET tracers to unveil additional features of vulnerable atherosclerotic lesions [104,105]. Immune checkpoints emerge as attractive imaging targets for detecting inflammatory and immune markers within the plaque microenvironment. Translational and human studies have explored immune-PET tracers employing molecules such as CD40-CD40L, CD80/86-CD28, and CD47 [106,107,108,109], even though this was conducted with mixed results [110]. Flores et al. conducted a study in a mouse model of atherosclerosis using immune-PET tracers for CD47. The authors showed that CD47 inhibition downregulated genes implicated in the macrophage’s response to IL-1 and IFN-γ, resulting in a significant reduction in atherosclerotic inflammation [67]. Magrolimab, a first-in-class anti-CD47 antibody, is currently used in clinical trials for hematological and solid malignancies [111]. Interestingly, an analysis of 13 non-Hodgkin’s lymphoma trial patients using 18F-FDG PET–CT after 9 weeks of Magrolimab treatment revealed a reduction in FDG signal uptake in most diseased carotid artery segments, suggesting the potential role of CD47 inhibition in reducing vascular inflammation [66].

These promising findings suggest the potential utility of molecular imaging in advancing our comprehension of atherosclerosis among patients undergoing ICI therapy. A heightened awareness of ICI-associated CV disease and the development of novel risk stratification tools are crucial for identifying individuals who stand to benefit most from preventive interventions [3]. However, the influence of traditional CV risk factors on ICI-associated atherosclerotic CV vents remains unclear. Studies have yielded conflicting results, with some indicating a heightened risk of CV events in patients with hypertension, dyslipidemia, prior CV disease, or subclinical atherosclerosis. Conversely, other investigations have surprisingly revealed no significant correlation between ICI-associated cardiovascular events and risk factors [3]. Indeed, further research efforts should be directed towards elucidating the impact of traditional CV risk factors on the development of CV adverse events in patients undergoing treatment with ICIs.

## 9. Conclusions

ICIs have ushered in a new era in oncology, emerging as the primary treatment modality for a wide spectrum of advanced malignancies. Their impact has been profound, yielding significant improvements in both progression-free survival (PFS) and OS across diverse patient populations. However, in recent years, the emergence of irAEs, with a particular focus on CV complications due to their potential severity, has raised concerns regarding the widespread use of ICIs. Additionally, mounting evidence suggests the potential association between ICI use and an increased susceptibility to plaque progression and ASCVD. Despite notable advances in understanding, the intricate interplay between ICIs and atherosclerotic diseases remains incompletely elucidated. The precise mechanisms by which ICIs may impact the atherosclerotic process at various stages continue to be actively investigated. From a clinical standpoint, studies conducted thus far are limited by inherent constraints, including small sample sizes and relatively short follow-up durations. Moreover, specific insights into the effects of ICIs on the CV system in both primary and secondary CV prevention settings are lacking. Similarly, there is a pressing need to explore therapeutic strategies for the prevention and treatment of atherosclerotic diseases in patients undergoing ICI therapy. Consequently, randomized clinical trials are eagerly awaiting to assess the safety of ICIs in this context and to meticulously evaluate the efficacy of CV protective drugs, such as statins and PCSK9 inhibitors, in preventing ICI-related side effects.

Given the increasing utilization of ICI therapies in clinical practice, coupled with the prevalence of CV risk factors that frequently coexist in cancer patients and the global significance of atherosclerotic diseases as a leading cause of morbidity and mortality, there is a critical need for comprehensive large-scale investigations to dissect the role of ICIs in atherogenesis, elucidate the impact of traditional CV risk factors on irAEs, and establish optimal preventive strategies and treatments to prevent the discontinuation of effective anti-cancer ICI therapies due to CV side effects. Given the variability in CV risk profiles among different cancer types and demographic cohorts, future randomized clinical trials should encompass diverse patient subtypes. This approach can facilitate a more comprehensive understanding of the complex interplay between risk factors, various malignancies, and the development of CV side effects associated with ICIs.

In conclusion, there is an imperative to strike a balance in cancer treatment, aiming to maximize therapeutic efficacy while simultaneously minimizing adverse effects. Achieving this outcome requires further studies to comprehensively understand the mechanisms underlying irAEs, along with adopting a multidisciplinary approach to patient management. The primary goal of this approach should be to ensure that the benefits of ICIs in terms of OS and PFS are not compromised by an elevated risk of CV morbidity and mortality.

## Figures and Tables

**Table 2:** Summary of clinical studies of ICI therapy and ASCVD.

Author	Study	Major Findings	ICI Effect
Hu et al. [9] (2017)	Meta-analysis (22 studies)	1.0% incidence of MI (95% CI:0–13%)	ASCVD RATE ↑
Bar et al. [75] (2019)	Single center retrospective	Single cohort: acute vascular events within 6 mo post-ICIs: 2.6% (95% CI: 1.8%–3.6%); event rate was 5.2% (95% CI: 2.8%–9.2%); acute vascular events incidence higher within 6 mo (OR: 3.49; 95% CI 1.45–8.41; *p* = 0.002). 1% of cases were MI or ischemic stroke. OS worse in post-Id patients with acute vascular events 3 mo vs. 14 mo; HR: 3.01; 95% CI:2–4.39; *p* < 0.0001	ASCVD RATE ↑
Wang et al. [76] (2019)	Meta-analysis (125 studies)	9.8% incidence of ASCVD rate treatment-related deaths due to CV irAEs, including MI, HF and CM	ASCVD RATE ↑
Nichetti et al. [78] (2020)	Prospective observational	6.5% incidence of acute vascular events (2 ACS, 9 strokes, 3 visceral arterial thromboses) within 16 mo	ASCVD RATE ↑
Oren et al. [77] (2020)	Single center retrospective	Rate of MI was 213 (7%) and stroke was 227 (7%) patients	ASCVD RATE↑
Drobni et al. [74] (2020)	Single center retrospective	Matched cohort, case crossover, and imaging study: higher risk of acute vascular events in ICIs (HR: 3.3; 95% Cl: 2.0– 5.5; *p* < 0.001) Case crossover: higher incidence of acute vascular events at 2 years after ICIs vs 2 years before ICIs (adjusted HR: 4.8; 95% CI: 3.6–6.5; *p* < 0.001) Imaging: Higher aortic plaque progression rate (2.1%/y before ICIs to 6.7%/y after ICIs)	ASCVD RATE andplaque ↑

ASCVD, atherosclerotic cardiovascular events; CM, cardiomiopathy; HF, heart failure; irAEs, immune-related adverse events; MI, myocardial infarction; mo, months. Up arrows indicate an increase in ASCVD rate or in plaque size.

## Data Availability

Not applicable.
